# Diagnostic value of metagenomic next-generation sequencing in patients with osteoarticular infections: a prospective study

**DOI:** 10.1128/spectrum.01064-24

**Published:** 2025-04-10

**Authors:** Qiang Zhang, Qiushi Wang, Feng Zhang, Xiuping Li, Yuhong Sun, Li Wang, Zhongfa Zhang

**Affiliations:** 1 Department of Orthopedics, Shandong Public Health Clinical Center, Jinan, China; 2 Infection Business Unit, Tianjin Novogene Med LAB Co., Ltd., Tianjin, China; 3 Department of Respiratory Medicine, Shandong Public Health Clinical Center, Jinan, China; London Health Sciences Centre, London, Ontario, Canada

**Keywords:** osteoarticular infections, primary, invasion, tuberculosis, infection, diagnosis

## Abstract

Due to the limits of traditional microbiological methods and the complexity of osteoarthritis pathogens, only a few pathogens can be detected. We evaluated metagenomic next-generation sequencing (mNGS) for detecting pathogens in osteoarthritis infection samples. We prospectively included 150 patients with osteoarthritis infection who visited the Orthopedics Department of Shandong Provincial Public Health Center from 2023 to 2024, including 124 cases of primary osteoarthritis (POI) and 26 cases of invasive osteoarthritis (IOI). The most common pathogenic bacteria were *Mycobacterium tuberculosis* complex, *Staphylococcus aureus*, and *Brucella melitensis*. mNGS (75.33%) significantly improved the detection of pathogens in osteoarticular infections compared to conventional tests (CT) methods (36.67%). mNGS could detect a wider spectrum of pathogens compared to CT methods, especially for mixed and rare pathogens. The treatment strategies for patients with osteoarthritis infection could be adjusted based on the results obtained from mNGS testing. Furthermore, the abundance of *M. tuberculosis* complex, *B. melitensis,* and *Staphylococcus epidermidis* was significantly correlated with clinical indicators.

**IMPORTANCE:**

Identifying the microorganisms responsible for osteoarthritis infection could help with early diagnosis and treatment. In this study, we compared the pathogen detection rate of metagenomic next-generation sequencing (mNGS) and CT methods in patients with osteoarthritis infection and found that mNGS had a higher microbial detection rate and a broader spectrum of pathogens (especially for mixed pathogens). This study demonstrates that mNGS is an ideal tool for detecting pathogens in patients with osteoarticular infections.

## INTRODUCTION

Osteoarticular infections mainly refer to bacteria or fungi propagating in bone tissue or joints and releasing toxins to destroy bone tissue or joints, causing local inflammatory reactions (1–3). Osteoarticular infections have a high recurrence and disabling, which not only increases the pain of patients and prolongs the length of hospital stay, but also seriously affects the prognosis of patients and the rehabilitation of patients (4). It has been reported that the most common pathogens in osteoarticular infections are *Mycobacterium tuberculosis* complex and *Staphylococcus aureus* (5, 6). Moreover, due to invasive medical intervention, the pathogenic spectrum of osteoarticular infection is quite broad (7–9), and some patients with polymicrobial infections (5) make diagnosis and treatment more difficult. Therefore, prompt and accurate microbiological testing is crucial in the diagnosis and therapy of osteoarticular infections.

Conventional microbial culture is the most common way to isolate and culture pathogenic bacteria. However, the conventional microbial culture has shortcomings, such as a poor positive rate and time-consuming processes (10). Metagenomic next-generation sequencing (mNGS) has been used in microbial identification as sequencing technology continues to evolve and improve. mNGS exhibits a large detection range and may identify bacteria, fungi, viruses, parasites, uncommon pathogens, and even undiscovered pathogens (11). Moreover, mNGS is an unbiased detection technology that can rapidly, efficiently, and accurately detect multiple pathogens across a large range (11, 12). mNGS has been applied to identify pathogenic microorganisms in many infectious diseases, such as pulmonary infections (13), sepsis (14), meningitis (15), and myocarditis (16). Previous studies have revealed that the detection rate of pathogens by mNGS is higher than that by traditional culture in patients with osteoarticular infections (92 cases) (17), spinal infection (38 cases) (18), invasive osteoarthritis (IOI, 92 cases) (5), periprosthetic joint infection (91 cases) (19), and TB spinal infection (37 cases) (20). However, these studies had limited sample numbers, a single kind of infection, and a narrow pathogen range. Furthermore, the laboratory environment, bioinformatic workflow, and sequencing platform are strongly correlated with mNGS results, which adds to the complexity of interpreting mNGS data (21).

In our study, we systematically evaluate the diagnostic value of mNGS in 150 osteoarticular infections and compare the pathogen detection rates using mNGS and conventional tests (CT) methods. Furthermore, we analyzed the correlation between clinical indicators and microbial species in osteoarticular infections. This study will provide a reference for improving the diagnosis and treatment of patients with osteoarticular infections.

## MATERIALS AND METHODS

### Study design and population

With the approval of the ethics committee of the Shandong Public Health Clinical Center, patients with suspected osteoarticular infection were prospectively enrolled in the Department of Orthopedics at the Shandong Public Health Center between 2023 and 2024. At least two senior orthopedic surgeons, two senior infectious diseases specialists, and one senior microbiologist confirmed the diagnosis together.

Inclusion criteria were as follows: (i) sufficient specimens for pathogen testing, including microbial culture and mNGS; (ii) patients who have complete medical records and agreed to participate in this study; and (iii) patients with suspected bone and joint infection based on the history of clinical manifestations and auxiliary examination. The exclusion criteria included (i) insufficient specimens for pathogenic bacteria detection; (ii) patients with incomplete cases; and (iii) HIV-infected patients.

### Specimen collection and microbial culture

Specimens, including tissues and pus were collected through surgery or CT-guided puncture. The tissue specimens were carefully placed in a sterile Eppendorf tube, followed by the addition of 1 mL of tryptic soy broth (HBPM0153, Haibo Biotechnology Co., Ltd., Qingdao, China). The tissue specimens were homogenized with an automatic rapid grinder (JXFSTPRP-24, Shanghai Jingxin Industrial Development Co., Ltd., Shanghai, China). Then, the homogenate was inoculated onto both anaerobic and aerobic blood agar plates for further analysis. In addition, Bactec Plus/F aerobic and anaerobic blood culture bottles (Becton-Dickinson, Germany) or BactecPeds Plus/F blood culture bottles (Becton-Dickinson, Germany) were inoculated with joint fluid and pus specimens and incubated for 14 days in a Bactec 9050 automated thermostat (Becton-Dickinson, Germany) (5).

### Conventional tests (CT)

CT for detecting pathogens in osteoarthritis infection samples used in this study included respiratory Pathogen 13-plex Test, fungal/bacterial culture and identification, Mycobacterial Culture and Identification for Tuberculosis, *Brucella* Antibody Screen (IgM and IgG), GeneXpert assay, T-SPOT test, and identification of *M. tuberculosis* (qPCR).

### mNGS

Using optimized procedures, we conducted mNGS on 5 pus, 4 joint fluid, and 141 tissue samples obtained from the 150 patients. The process of mNGS is briefly described as follows: (i) nucleic acid extraction: the total deoxyribonucleic acid (DNA) was extracted from liquid or homogenate using the Novogene PD-seq metagenomic DNA extraction kit (PT103, Tianjin Novogene Medical, Tianjin, China) following the provided instructions. (ii) Library construction and sequencing: DNA libraries were constructed using the Novogene PD-seq metagenomic DNA library kit (PT104, Tianjin Novogene Medical, Tianjin, China). The DNA was digested into 250–350 bp fragments using the enzyme. Subsequently, the fragments were repaired, end-performing phosphorylation was carried out, adaptors were ligated, and a polymerase chain reaction (PCR) amplification step was performed for further amplification. After purifying the PCR product, libraries were pooled and sequenced for 50 bp single ends using a GenoLab M (GeneMind Biosciences Co., Ltd., Shenzhen, China) equipment. The average data output from samples was 50 million reads.

### Bioinformatics analysis

Junctions and low-quality or length <15 bp reads were filtered out using fastp (version 0.20.1). Bowtie2 (version 2.3.5.1) was employed to align the human reference genome (GRCH38+YHref) to identify and remove human sequencing data. After eliminating human reads, the remaining reads were aligned with our microbial genome database (PD-seq database, version 3.0), and their genome sequences were obtained from RefSeq, GenBank, and NT databases. The database utilized in this study consists of 11,637 bacteria, 12,229 viruses, 4,158 fungi, and 876 parasites. Species identification was conducted by mapping the microbial database using the Kraken2+Bracken workflow. Sequence reads were annotated using Kraken2 (version 2.1.2), and species abundance was estimated using Bracken software (version 2.6). The methods for determining true positive results are as follows: (i) the number of specific sequences of clinically reported pathogenic bacteria (CRP) or non-CRP in patient samples can be more than three times the reading of negative controls, and the relative abundance of non-CRP should exceed 10%; (ii) the sequence of *M. tuberculosis* in the patient should be greater than 1; (iii) considering the impact caused by cross-contamination or spillover, the true positive species detected in the same batch should be 1/1,000 or more than the strong positive (more than 1,000 specific sequences) (22); (iv) the internal control (Spike-in-control) (ZYMO Research, Irvine, CA, USA) spiked more than 200 reads in the negative control, and all samples were detected; (v) species with a detection frequency of more than 25% in the negative control are considered environmental pollutants and will be removed; and (vi) species colonizing the skin were filtered into the microecology in the final report. For suspected positive pathogens initially screened using the above steps, blast validation is performed for positive pathogens, and samtools is used to obtain coverage and average depth.

### Clinical composite diagnosis

The clinician determined whether the patient had an osteoarthritis infection according to the Chinese guideline for diagnosis and treatment of osteoarthritis (2021 edition). The causative pathogens are then determined based on microbiological examination (including mNGS and CT methods), imaging, pathology, and treatment response. Following a thorough discussion, clinicians, laboratory doctors, and seasoned mNGS report interpretation specialists made the ultimate decision.

### Pearson correlation analysis

Pearson correlation coefficient and corresponding *P* value between clinical indicators and the pathogenic bacteria of osteoarticular infections were calculated using the WGCNA function package (23) and visualized using the R language “pheatmap” function. The correlation between inflammation indicators and microbial DNA abundance was analyzed using the R language “cor” function, and the result was visualized using the “ggplot” function.

### Statistical analysis

Statistical analysis of the mean ± standard deviation (SD) was used to represent the normal distribution of numerical variables. Percentages are used to represent categorical data. The chi-square test and *t* test were used for the comparative analysis of categorical and numerical variables, respectively. *P* < 0.05 was considered statistically significant.

## RESULTS

### Demographic and clinical features of patients

A total of 150 patients with osteoarticular infections were included in this study. We collected puncture tissue samples from 141 patients, pus samples from 5 patients, and pus from 4 patients. Among these 150 patients, 124 patients were diagnosed with primary osteoarthritis (POI) and 26 patients were diagnosed with IOI. The demographic and clinical characteristics of the patients are presented in Table 1. There were no meaningful discrepancies in age, gender, and blood cell count between POI and IOI patients. In the POI and IOI groups, 24 patients had a history of diabetes, and 17 patients had a history of hypertension.

**TABLE 1:** The clinical features of patients with osteoarticular infections^*a*^

Clinical characteristics	Total (*n* = 150)	POI (*n* = 124)	IOI (*n* = 26)	*P*
Age, mean (SD), years	58.29 (14.33)	58.45 (14.50)	58.28 (14.33)	0.99
Female, *n* (%)	69 (46)	62 (50)	7 (26.92)	0.96
Sample type				
Pus, *n* (%)	5 (3.33)	4 (3.23)	1 (3.85)	0.27
Joint fluid, *n* (%)	4 (2.67)	3 (2.42)	1 (3.85)	0.42
Tissue, *n* (%)	141 (94.00)	117 (94.35)	24 (92.31)	0.99
Site of infection				
Lower limb bones, *n* (%)	33 (22.00)	23 (18.55)	10 (38.46)	0.01
Upper limb bones, *n* (%)	13 (8.67)	12 (9.68)	1 (3.85)	0.01
Vertebra, *n* (%)	103 (68.67)	88 (70.97)	15 (57.69)	0.47
Bone of the thorax, *n* (%)	1 (0.67)	1 (0.81)	0 (0.00)	0.68
Clinical symptoms				
Fever, *n* (%)	45 (30.00)	37 (29.84)	8 (30.77)	0.99
Loss of weight, *n* (%)	30 (20.00)	23 (18.55)	7 (26.92)	0.40
Sweats	6 (4.00)	6 (4.84)	0 (0.00)	0.10
Blood cell count				
White blood cell, mean (SD)	5.85 (1.91)	5.90 (1.95)	5.83 (1.92)	0.99
NEUT (%), mean (SD)	62.93 (17.26)	63.16 (17.94)	62.63 (17.32)	0.99
Lymphocyte (%), mean (SD)	23.00 (10.24)	21.90 (10.82)	22.92 (10.28)	0.98
Hemoglobin (g/L), mean (SD)	115.99 (20.26)	115.60 (20.21)	115.36 (20.33)	0.99
CRP (mg/L), mean (SD)	42.77 (53.77)	42.08 (54.91)	42.58 (53.86)	0.99
ESR (mm/h), mean (SD)	50.42 (32.84)	50.09 (31.58)	50.30 (32.95)	0.99
Medical history				
Diabetes, *n* (%)	24 (16)	18 (14.52)	6 (23.08)	0.31
Immunosuppression, *n* (%)	4 (2.67)	4 (3.23)	0 (0)	0.22
Hepatic dysfunction, *n* (%)	2 (1.33)	2 (1.61)	0 (0)	0.47
High blood pressure, *n* (%)	17 (11.33)	12 (9.68)	5 (19.23)	0.14

^*a*^ CRP, C-reactive protein; ESR, erythrocyte sedimentation rate; IOI, invasive osteoarthritis; NEUT, neutrophilic granulocyte percentage; POI, primary osteoarthritis.

### Pathogen composition

**Fig 1:**
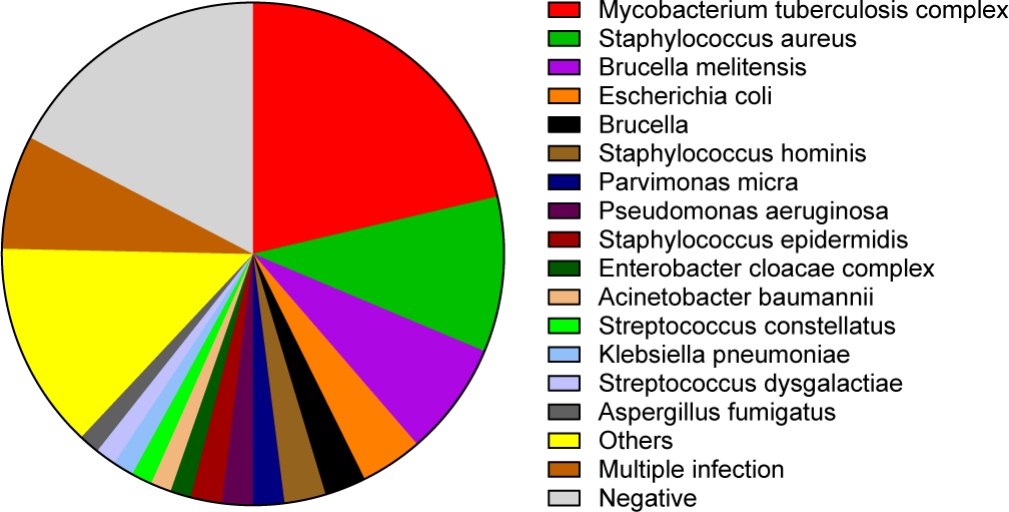
The pathogenic bacteria spectrum of osteoarticular infection.

In this study, pathogens were detected in 150 patients with osteoarticular infections using mNGS and various traditional pathogen detection methods. As shown in Fig. 1, in the 150 patients with osteoarticular infections, the most common pathogenic bacteria were *M. tuberculosis complex* (32 cases), *S. aureus* (15 cases), and *Brucella melitensis* (11 cases) (Fig. 1). Many rare pathogens, such as *Staphylococcus lugdunensis*, *Mycobacterium marinum*, *Vagococcus fluvialis*, and *Neisseria subflava*, were detected in POI patients (Fig. S1).

### Comparison of the diagnostic performances of mNGS and CT methods

**Fig 2:**
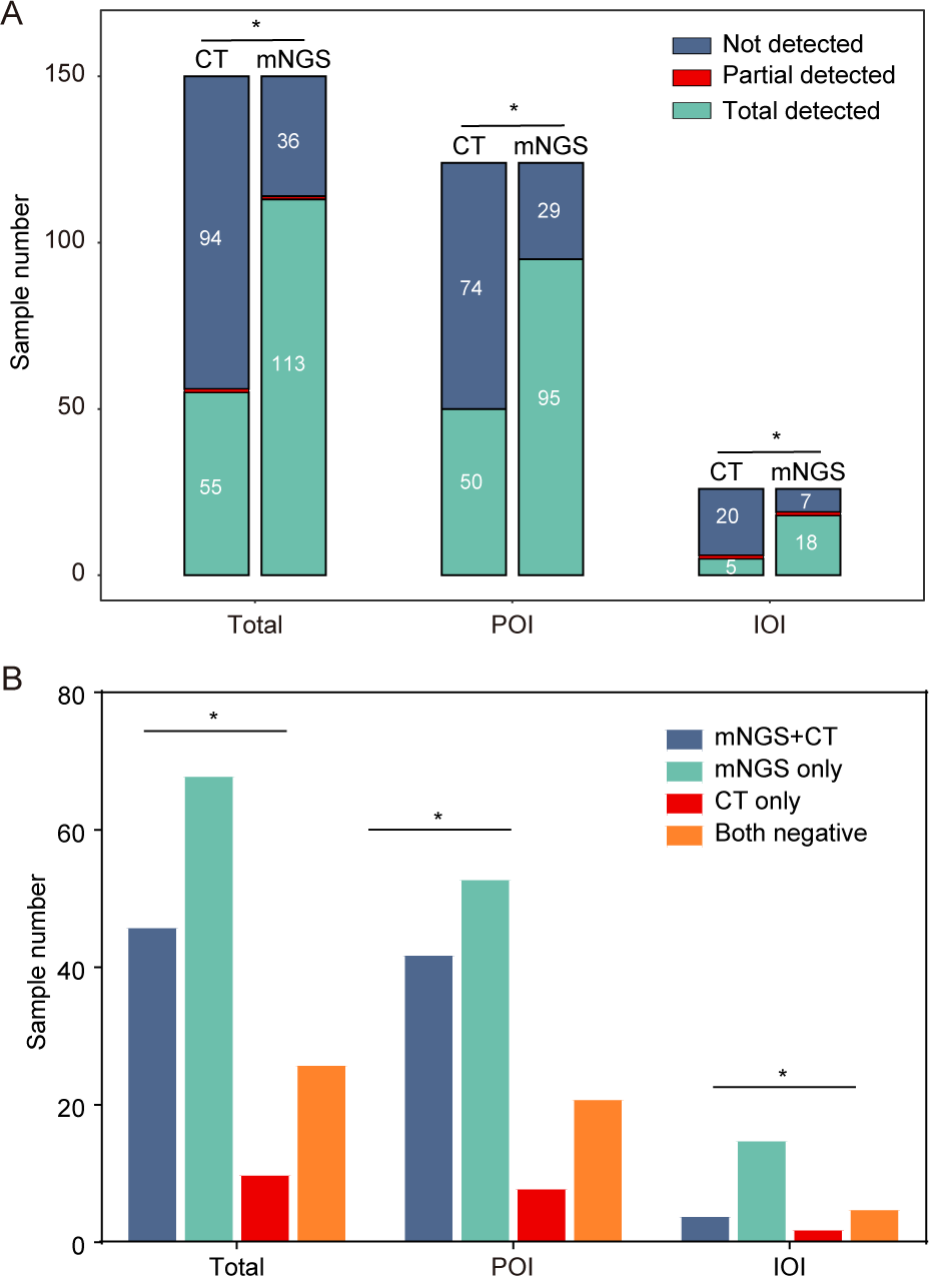
Comparison of the diagnostic performances of metagenomic next-generation sequencing (mNGS) and conventional tests (CT) methods. (**A**) Number of samples with total detected, partially detected, and no detected pathogens using mNGS and CT methods. (**B**) Number of samples in which pathogens were detected by only mNGS, both the mNGS and CT methods, and CT method only. *p < 0.05.

Taking the final clinical diagnosis as a reference, we evaluated the performance of mNGS and CT methods for pathogen identification. Among 150 patients, the positive rate of mNGS (75.33%) was significantly higher than that of the CT methods (36.67%). The sample number of mNGS (24%) that did not detect any pathogen was significantly lower than that of the CT methods (62.67%). The percentage of mNGS-positive cases was significantly higher than that of CT-positive cases in POI and IOI groups (Fig. 2A). Moreover, we found that pathogenic bacteria in the majority of patients were identified using only mNGS (45.33%) or simultaneous mNGS and CT methods (30.67%) (Fig. 2B). In contrast, only a limited number of patients had pathogens detected solely through CT methods (6.67%) (Fig. 2B).

### Comparison of pathogens detected using the mNGS and CT methods

**Fig 3:**
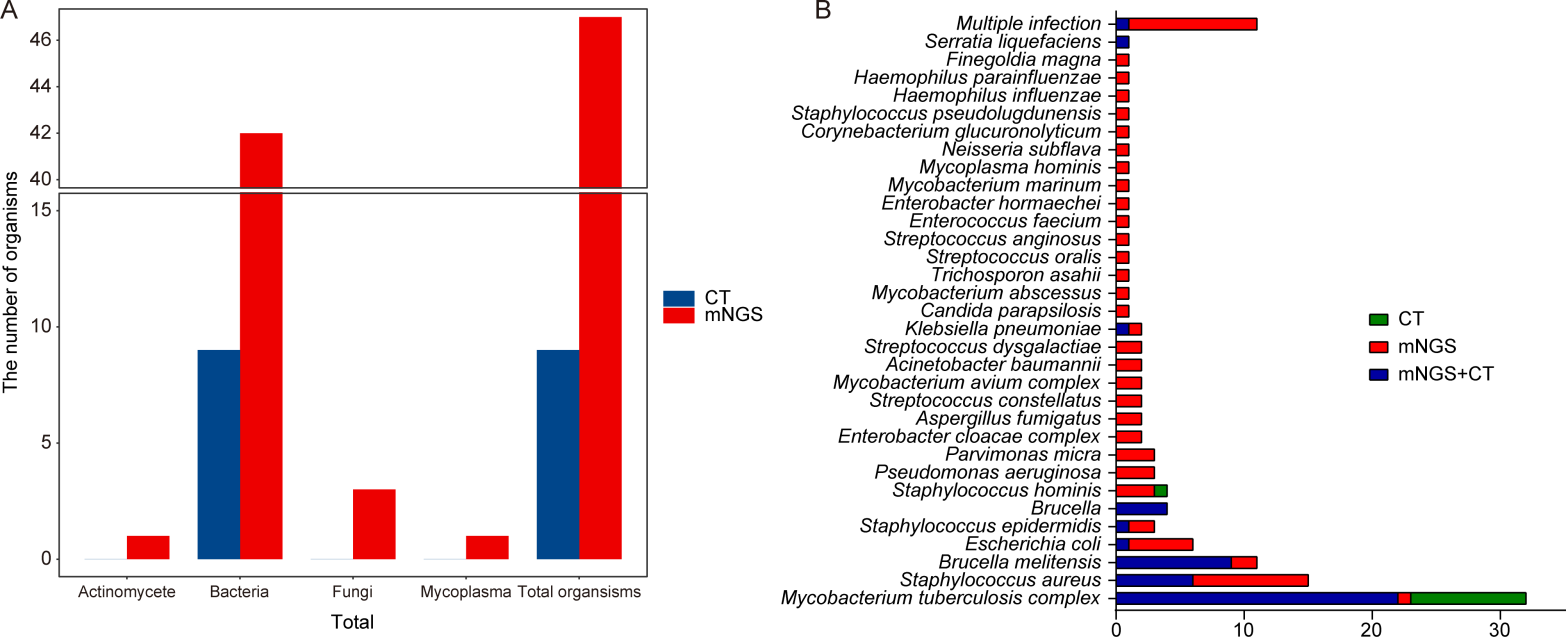
Comparison of pathogens detected using the mNGS and CT methods. (**A**) Comparison of the type of organisms detected using the mNGS and CT methods. (**B**) Pathogens identified using both the mNGS and CT methods, mNGS only, and CT method only. CT, conventional tests; mNGS, metagenomic next-generation sequencing.

As shown in Fig. 3A, a total of 47 organisms were identified using mNGS, while 9 organisms were identified using the CT method in 150 patients with osteoarticular infections. Compared to the CT method, mNGS identified more bacteria (42 vs 9), fungi (3 vs 0), Actinomycete (1 vs 0), and Mycoplasma (1 vs 0). Similarly, in POI and IOI groups, mNGS also identified more organisms than the CT method (Fig. S2). In 150 patients with osteoarticular infections, the pathogen with the highest detection rate by mNGS or CT was *M. tuberculosis* complex, followed by *S. aureus* and *B. melitensis*. Except for that of *M. tuberculosis* complex and *Brucella*, the positive detection rate of various pathogens of mNGS was higher than that of the CT method, especially for mixed pathogens (Fig. 3B). These findings suggested that the pathogen spectrum detected by mNGS was significantly broader than that of the CT method, which was also observed in POI and IOI patients (Fig. S3).

In addition, the number of reads per sample was 8.06–99.02 million for mNGS. Next, we analyzed the reader number and reads per million (RPM) of top 11 pathogens. As shown in Fig. S4A, the pathogen with the highest reader number was *Escherichia coli*, followed by *B. melitensis* and *S. aureus*. The pathogen with the highest RPM was *M. tuberculosis* complex, followed by *E. coli* (Fig. S4B).

### Performance of mNGS and CT detection methods in patients with tuberculosis infection

**Fig 4:**
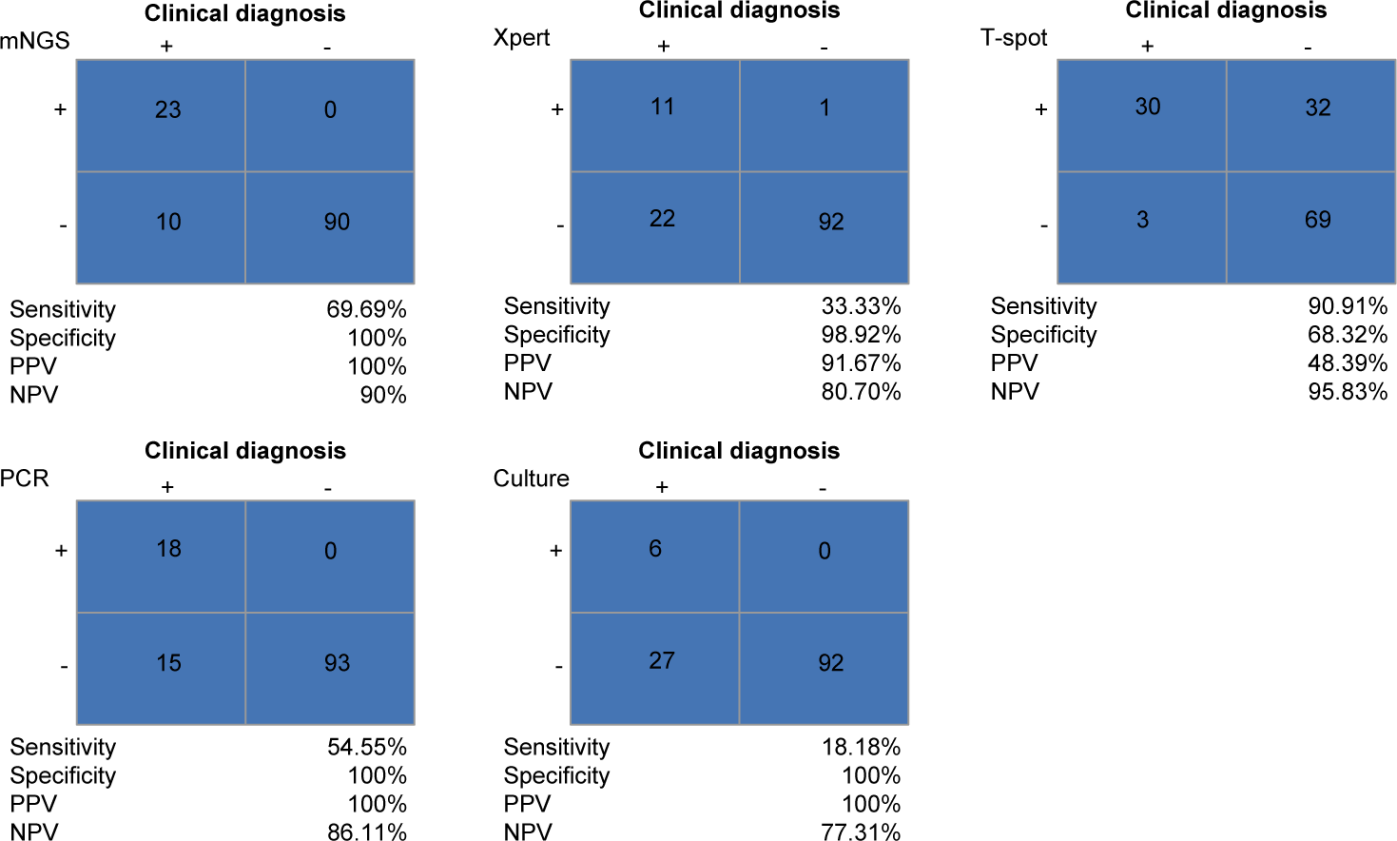
The diagnostic performance of mNGS and CT methods in identifying suspected TB cases. CT conventional tests; mNGS, metagenomic next-generation sequencing.

Our above study showed that the *M. tuberculosis* complex was the most frequently detected pathogen in osteoarthritis infections. Therefore, we next analyzed the diagnostic performance of mNGS and CT methods for tuberculous osteoarthritis with the final comprehensive clinical diagnosis as the gold standard. As shown in Fig. 4, the T-SPOT method (90.91%) had the highest sensitivity in detecting *M. tuberculosis*, followed by mNGS (69.69%), qPCR (54.54%), GeneXpert (33.33%), and culture (18.18%). The specificity of mNGS (qPCR or culture) was significantly higher than that of the T-SPOT method (100% vs 68.32%, *P* < 0.0001). The PPV of mNGS for identifying *M. tuberculosis* was significantly higher than the T-SPOT method (100% vs 48.39%, *P* < 0.0001). Moreover, there was no significant difference in NPV among these methods. These results suggested that mNGS had higher accuracy in diagnosing TB patients.

### Influence of mNGS diagnosis on medication of patients

**Fig 5:**
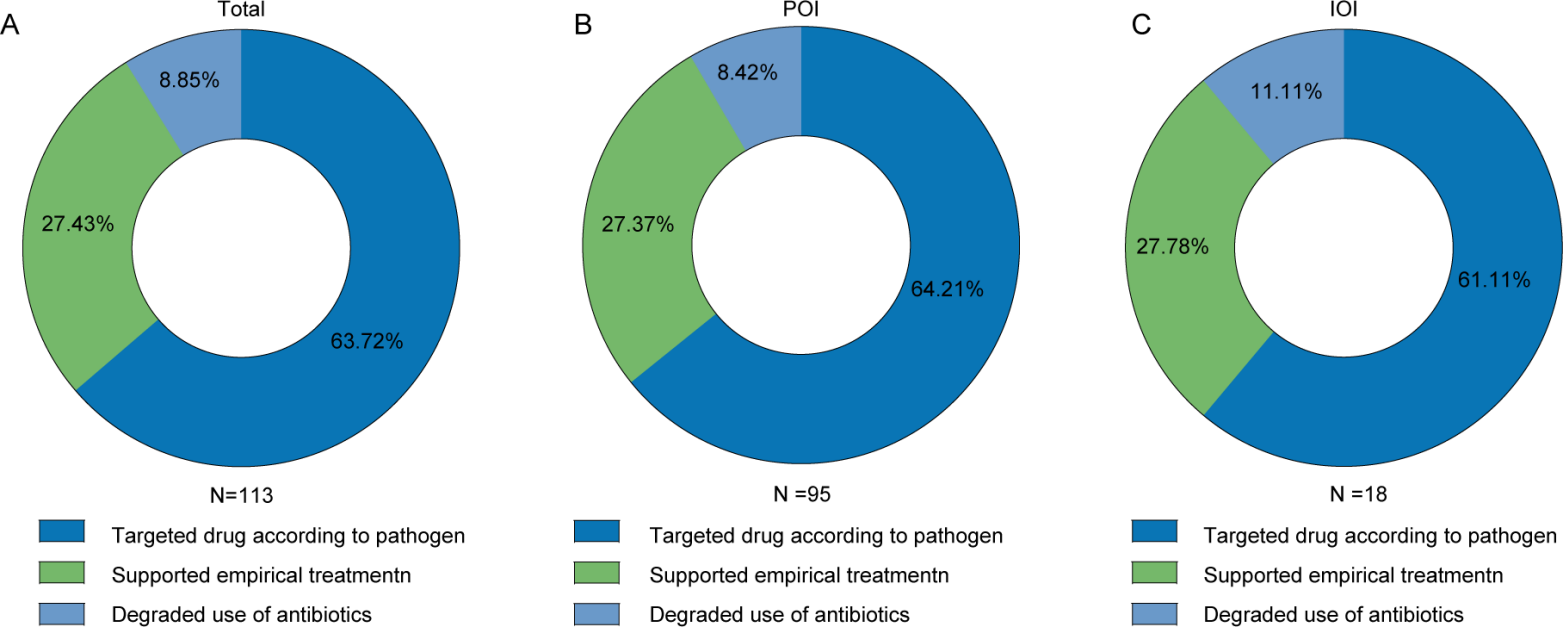
Clinical impact of mNGS positive results. (**A**) Total osteoarticular infections group. (**B**) POI group. (**C**) IOI group. IOI, invasive osteoarthritis; POI, primary osteoarthritis.

Among the 113 mNGS-positive patients, 72 (63.72%) patients adjusted targeted drugs according to pathogens, 31 (27.43%) patients had no change in treatment, supporting the original experiential treatment, and 10 (8.85%) patients adjusted antibiotics (degraded) (Fig. 5A). In the POI and IOI groups, most patients adjusted their targeted medication, and some patients reduced their antibiotic use based on the pathogens detected by mNGS (Fig. 5B and C). These results indicated that treatment strategies for patients with osteoarthritis infection can be adjusted based on the test results of mNGS.

### The correlation between clinical indicators and microbial DNA abundance

**Fig 6:**
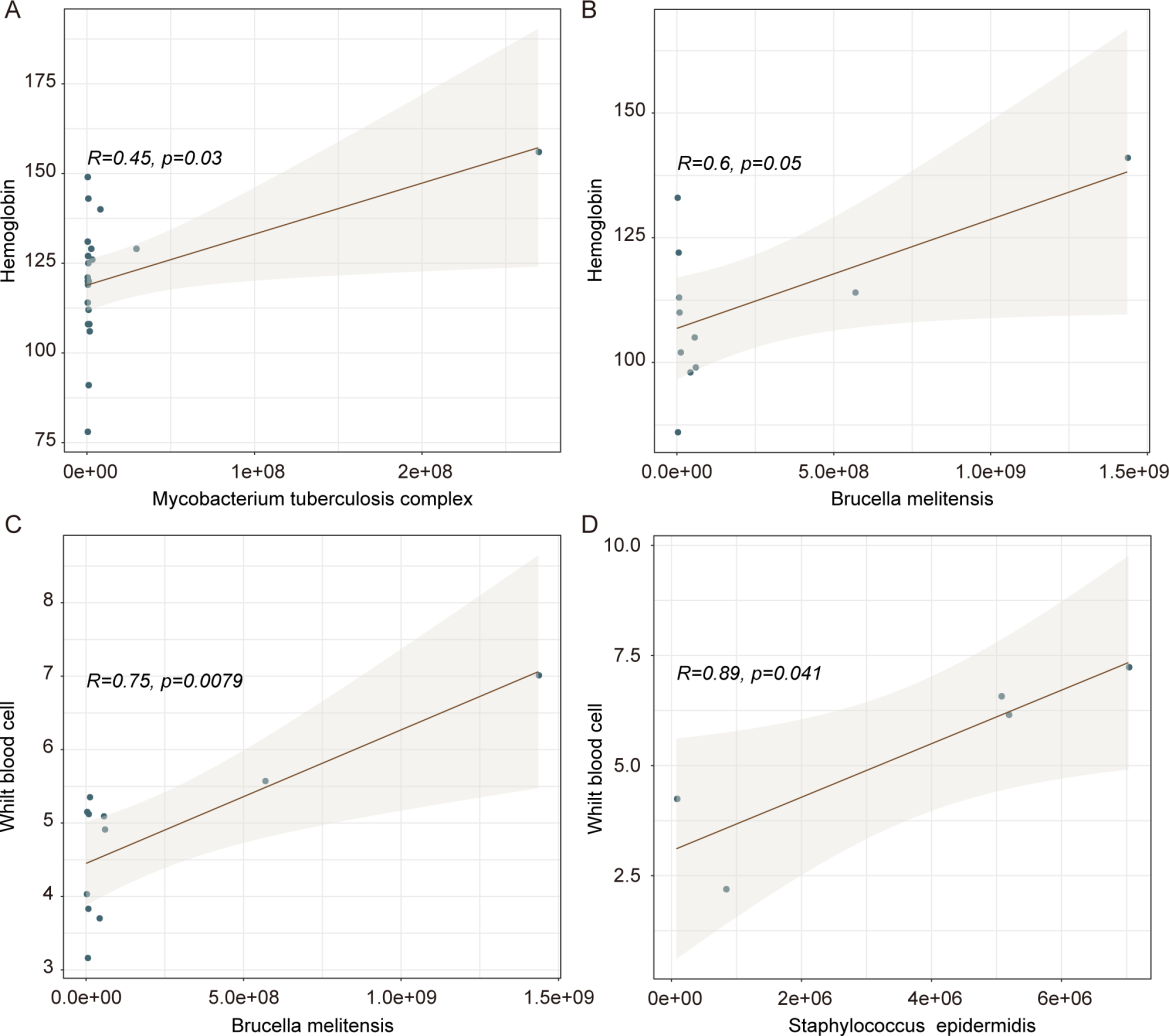
The correlation between clinical indicators and microbial DNA abundance. The correlation between hemoglobin and *M. tuberculosis* complex (**A**), hemoglobin and *B. melitensis* (**B**), WBC and *B. melitensis* (**C**), WBC and *S. epidermidis* (**D**). WBC, white blood cells.

Furthermore, we performed Pearson to analyze the correlation between clinical indicators and DNA abundance of the top 10 microbial species in patients with osteoarticular infections. We found that hemoglobin was significantly positively correlated with the DNA abundance of *M. tuberculosis* complex and *B. melitensis* (Fig. 6A and B). Additionally, WBC exhibited a significant positive association with the DNA abundance of *B. melitensis* and *S. epidermidis* (Fig. 6C and D).

## DISCUSSION

Orthopedic patients are prone to pathogen infection due to open trauma, soft tissue injury, the treatment of invasive surgery, the use of various internal implants, long-term bedridden, and poor local blood supply (24–26). A better understanding of microorganisms in patients with osteoarticular infections is conducive to early diagnosis and treatment and will improve the prognosis of patients. mNGS has been used clinically for pathogenic testing of infectious diseases, such as respiratory infections (27) and hematologic infections (28). A growing number of studies confirmed that mNGS had higher sensitivity and specificity than traditional microbial culture in diagnosing infectious diseases (18, 29). In the present study, we compared the pathogen detection rate of mNGS and CT methods in patients with osteoarthritis infection. Our findings indicated that mNGS exhibited great potential in the diagnosis of osteoarthritis infection, improving diagnostic accuracy and speed. Furthermore, we discovered that treatment strategies for patients with osteoarthritis infection can be adjusted based on the results obtained from mNGS testing, which has a potential impact on treatment outcomes.

In this study, the most prevalent pathogenic bacteria identified in patients with osteoarticular infections were the *M. tuberculosis* complex, *S. aureus*, and *B. melitensis*. These three microorganisms are commonly associated with osteoarticular infections (5, 6, 30). Fang et al. reported that *S. aureus* was the most frequently identified pathogen in POI and IOI patients, while *M. tuberculosis* ranked as the second most common pathogen in POI (5). Zhang et al. found that the most frequently detected species through mNGS in patients with spinal infections were *M. tuberculosis* complex and *S. aureus* (18). Additionally, Huang et al. utilized mNGS and culture methods to detect pathogens in 92 osteoarticular infection patients, revealing that *coagulase-negative Staphylococci* were the most commonly identified genus, followed by *S. aureus* (17). These findings underscore the significance of *M. tuberculosis* complex and *S. aureus* as major contributors to osteoarticular infections.

mNGS demonstrated a higher microbial detection rate and a broader spectrum of pathogens in patients with osteoarticular infections, particularly in cases involving mixed pathogens. In children with community-acquired pneumonia, the total positive coincidence rate was 86.78% for mNGS compared to 66.94% for conventional culture techniques (31). Among 82 abscess samples from patients with osteoarticular infections, mNGS identified more bacteria, mycobacterial, and fungal pathogens compared to the CT method, which was consistent with our results (6). In a study involving 408 sonicated fluid samples from prosthetic joint infections, mNGS identified 94.8% of known pathogens and 9.6% of potential pathogens in culture-positive cases, and 43.9% of new potential pathogens in culture-negative cases (32). Furthermore, in culture-positive acute spinal infection samples, mNGS identified approximately 93.75% of the pathogens (33). Moreover, Huang et al. reported that among 130 osteoarticular infection samples, mNGS identified 12 pathogenic strains that were undetected by microbiological culture (17). This evidence suggested that the sensitivity of mNGS for diagnosing osteoarticular infections was superior to that of CT methods, which might help the clinic diagnosis. However, investigations in more patients from different medical institutions are also needed to further evaluate the diagnostic value of mNGS.

Osteoarticular TB is a frequent kind of extrapulmonary TB, accounting for 15–20% of TB cases in Asia. Spinal TB accounts for around 50% of bone TB cases (34). Taking the final clinical diagnosis as the gold standard, we found that mNGS detected less *M. tuberculosis* compared with the CT method. Previous studies have also reported the limitations of mNGS in the detection of *M. tuberculosis*. Chen et al. have reported that five patients with lower respiratory tract infections were diagnosed with tuberculosis via conventional detection, while mNGS detected only one case of them (35). In an osteoarticular TB patient, only 9 of the 32, 990,757 mNGS readings from synovial fluid matched the *M. tuberculosis* genome (36), which might be due to the lower concentration of DNA extracted from *M. tuberculosis. M. tuberculosis* is a bacterium with a thick cell wall, and it is difficult to break the wall and release DNA, which may be the reason for the low efficiency of DNA extraction. Although the physical and chemical wall-breaking method was adopted in the sample processing, the content of *M. tuberculosis* was low, which did not reach the detection limit of mNGS (LOD: 100–1,000 copies/mL), resulting in the presence of a false negative. It has been reported that using early lipid cultures of specimens and enriching the low-concentration of *M. tuberculosis* DNA by biotinylated-RNA bait hybridization can improve the sensitivity of mNGS for *M. tuberculosis* detection (37, 38). Accordingly, when mNGS is performed on the samples of osteoarticular infection patients suspected of *M. tuberculosis* infection, it is necessary to optimize the DNA extraction process or enrich the DNA to improve the detection sensitivity. Some studies indicated that the T-SPOT test had diagnostic value for active TB; however, it exhibited low specificity for TB infection (20, 39), which was confirmed by our findings that the T-SPOT method had higher sensitivity and lower specificity in detecting *M. tuberculosis* compared to mNGS. These findings suggested that mNGS had higher accuracy in diagnosing TB patients. The combination of mNGS and T-SPOT detection can improve the diagnostic efficiency of TB.

mNGS can identify pathogens and guide doctors in adjusting the treatment program and observing the effect (40). Our results showed that treatment strategies for patients with osteoarthritis infection can be adjusted based on the results obtained from mNGS testing. Moreover, after adjusting the medication, the condition of patients improved. Therefore, the detection results of mNGS for pathogens had better clinical utility. Moreover, this study revealed a noteworthy positive correlation between hemoglobin levels and the DNA abundance of the *M. tuberculosis* complex and *B. melitensis*. This suggested that higher hemoglobin concentrations might be associated with an increased presence of these bacterial pathogens. Furthermore, the study also observed a significant positive association between white WBC counts and the DNA abundance of *B. melitensis* and *S. epidermidis*. This correlation could imply that the body’s immune response, as indicated by elevated WBC levels, was linked to the proliferation of these bacteria. These insights could potentially inform the development of diagnostic strategies and therapeutic approaches for osteoarthritis infections.

Nevertheless, there are several limitations to this study. There may be potential false positives and inherent contamination risks in the mNGS workflow. It mainly includes microorganisms or nucleic acids introduced during sample collection and transportation, normal colonizing microorganisms, background microorganisms during detection, cross-contamination between samples, and species identification errors. Minimize false positives by following these steps. During sample collection and transportation, aseptic operation is ensured to reduce the introduction of external microorganisms. In the experiment, negative controls were used, the background microorganisms were filtered out by our curated background database, and the double-ended UDI index was used to reduce index hopping. At the same time, the filtering conditions of raw data are used to reduce the cross-contamination of strong positive samples. By establishing a colonization database of different sample types, colonizing microorganisms were defined as microecology. The false positives of comparison were reduced by blast validation. In addition, this study was conducted in a single center and included a total of 150 samples, focusing on POI and IOI. Therefore, larger multicenter studies covering different types of infections are needed.

### Conclusion

In conclusion, mNGS is a rapid and effective diagnostic method for osteoarthritis infections. Compared to the CT method, mNGS demonstrated a higher microbial detection rate and a broader spectrum of pathogens, particularly in identifying mixed and rare pathogens, thereby reducing the likelihood of missed or misdiagnoses. Additionally, our findings indicated that treatment strategies for patients with osteoarthritis infections could be tailored based on mNGS test results. However, mNGS also has certain limitations and should be used in conjunction with traditional microbial detection methods.

## Data Availability

The raw sequence data reported in this paper have been deposited in the Genome Sequence Archive (Genomics, Proteomics & Bioinformatics 2021) in National Genomics Data Center (Nucleic Acids Res 2022), China National Center for Bioinformation/Beijing Institute of Genomics, Chinese Academy of Sciences (GSA CRA015062) that are publicly accessible at https://ngdc.cncb.ac.cn/gsa. The BioProject is PRJCA023686.

## SUPPLEMENTAL MATERIAL

Figure S1 spectrum.01064-24-s0001.tif The pathogenic bacteria spectrum of primary (A) and invasive (B) osteoarticular infection.

Figure S2 spectrum.01064-24-s0002.tif Comparison of the type of organisms detected using the mNGS and CT methods in POI (A) and IOI (B) group.

Figure S3 spectrum.01064-24-s0003.tif Pathogens identified using both the mNGS and CT methods, mNGS only, and CT method only in POI (A) and IOI (B) groups.

Figure S4 spectrum.01064-24-s0004.tif The read number and reads per million (RPM) of top 11 pathogens in 150 samples.

Legends spectrum.01064-24-s0005.docx Legends for supplemental figures.

ASM does not own the copyrights to Supplemental Material that may be linked to, or accessed through, an article. The authors have granted ASM a non-exclusive, world-wide license to publish the Supplemental Material files. Please contact the corresponding author directly for reuse.
