# Supplementary figures and images for: Diagnostic value of metagenomic next-generation sequencing in patients with osteoarticular infections: a prospective study
Source: Microbiol Spectr. 2025 Apr 10;13(5):e01064-24. doi: 10.1128/spectrum.01064-24 (PMC12054075; doi:10.1128/spectrum.01064-24)

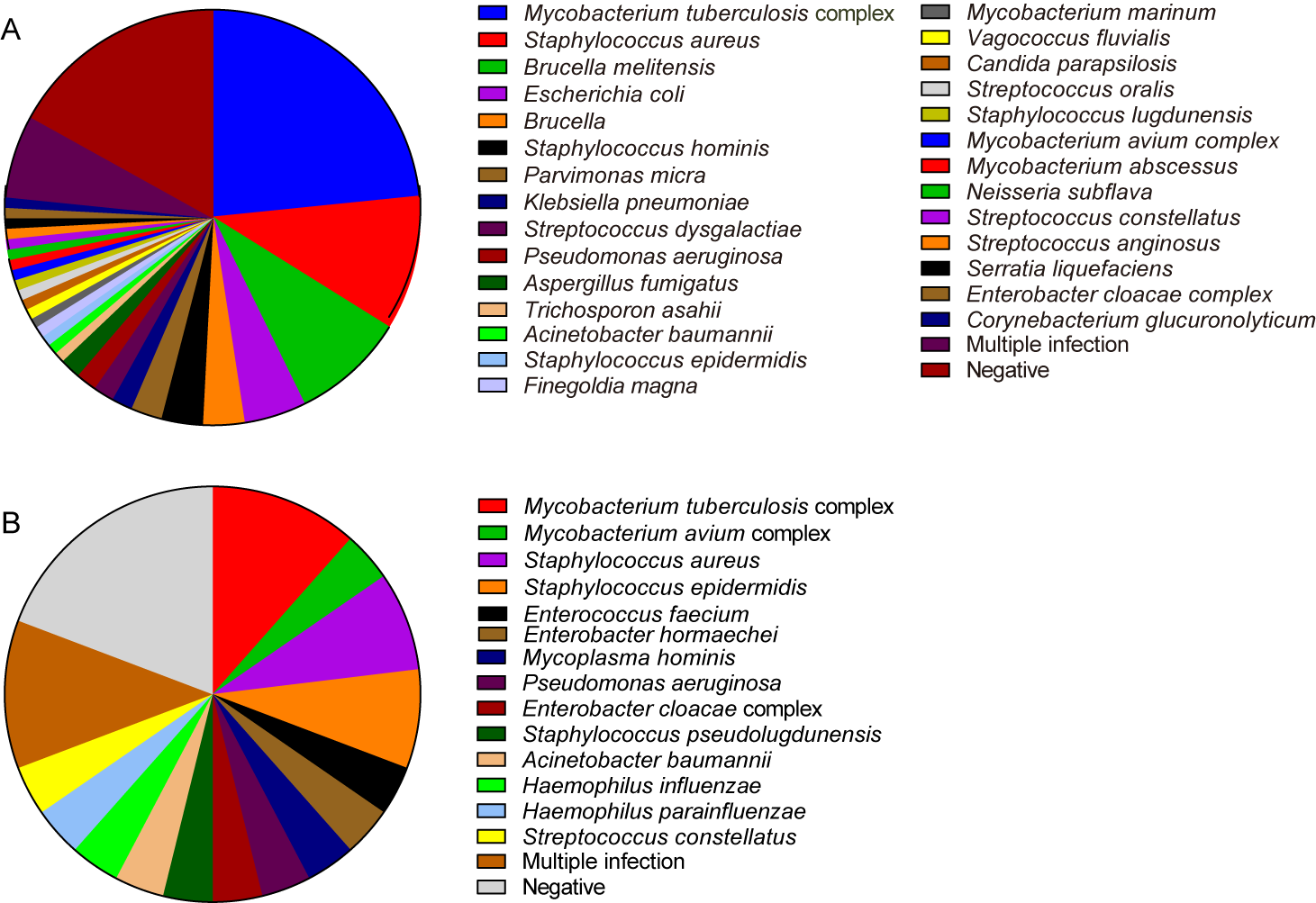

Supplement: Figure S1 — The pathogenic bacteria spectrum of primary (A) and invasive (B) osteoarticular infection. [file spectrum.01064-24-s0001.tif]

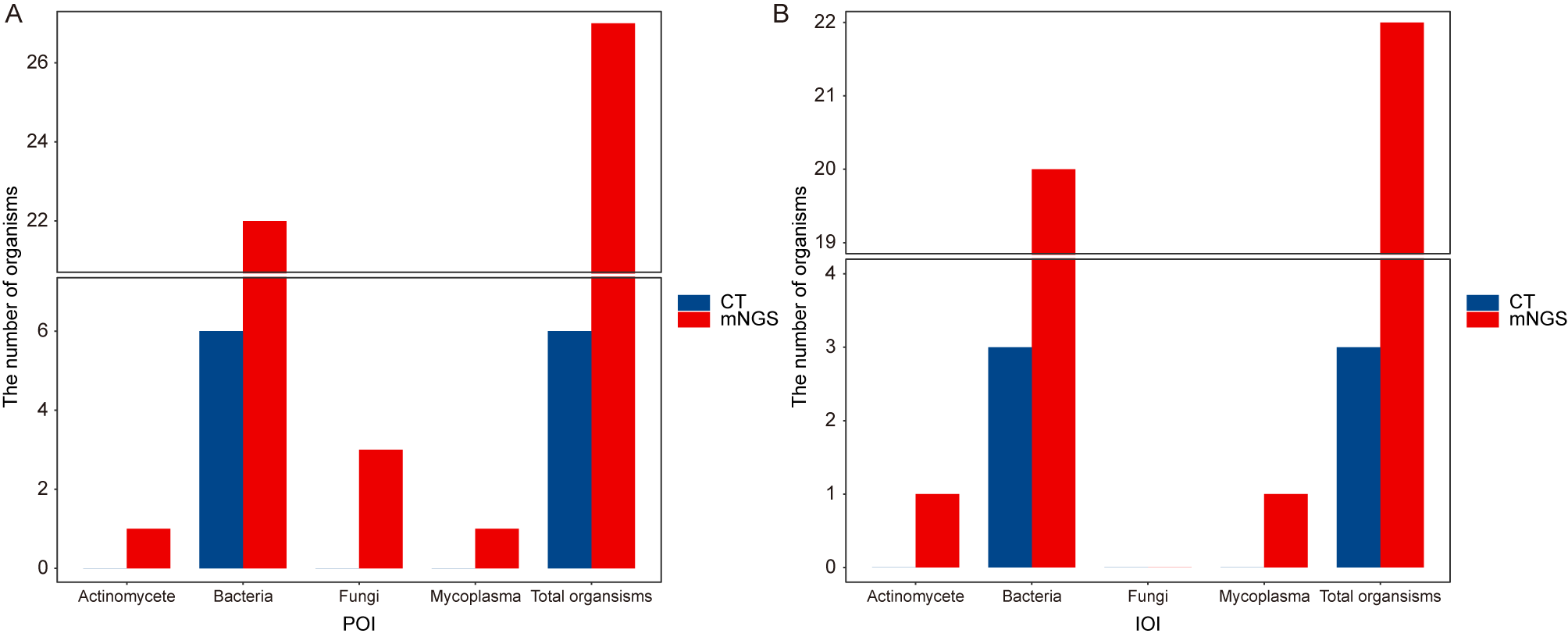

Supplement: Figure S2 — Comparison of the type of organisms detected using the mNGS and CT methods in POI (A) and IOI (B) group. [file spectrum.01064-24-s0002.tif]

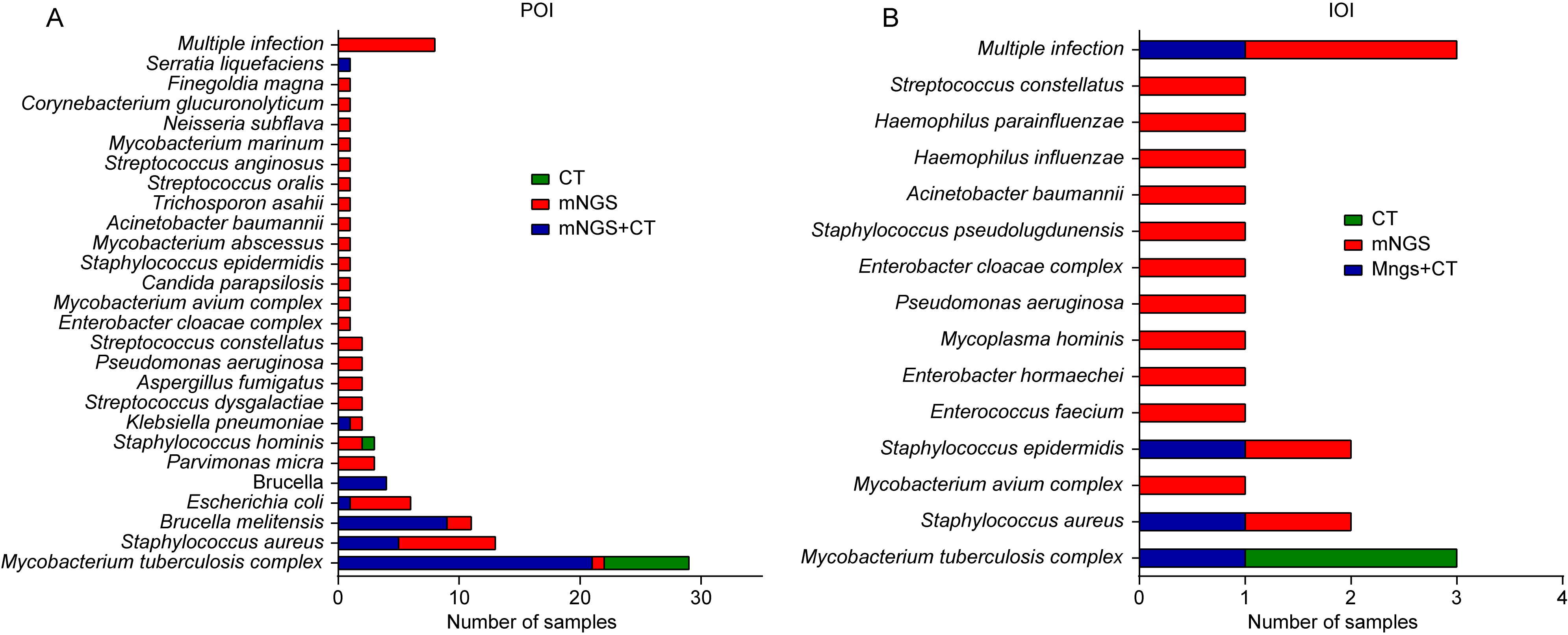

Supplement: Figure S3 — Pathogens identified using both the mNGS and CT methods, mNGS only, and CT method only in POI (A) and IOI (B) groups. [file spectrum.01064-24-s0003.tif]

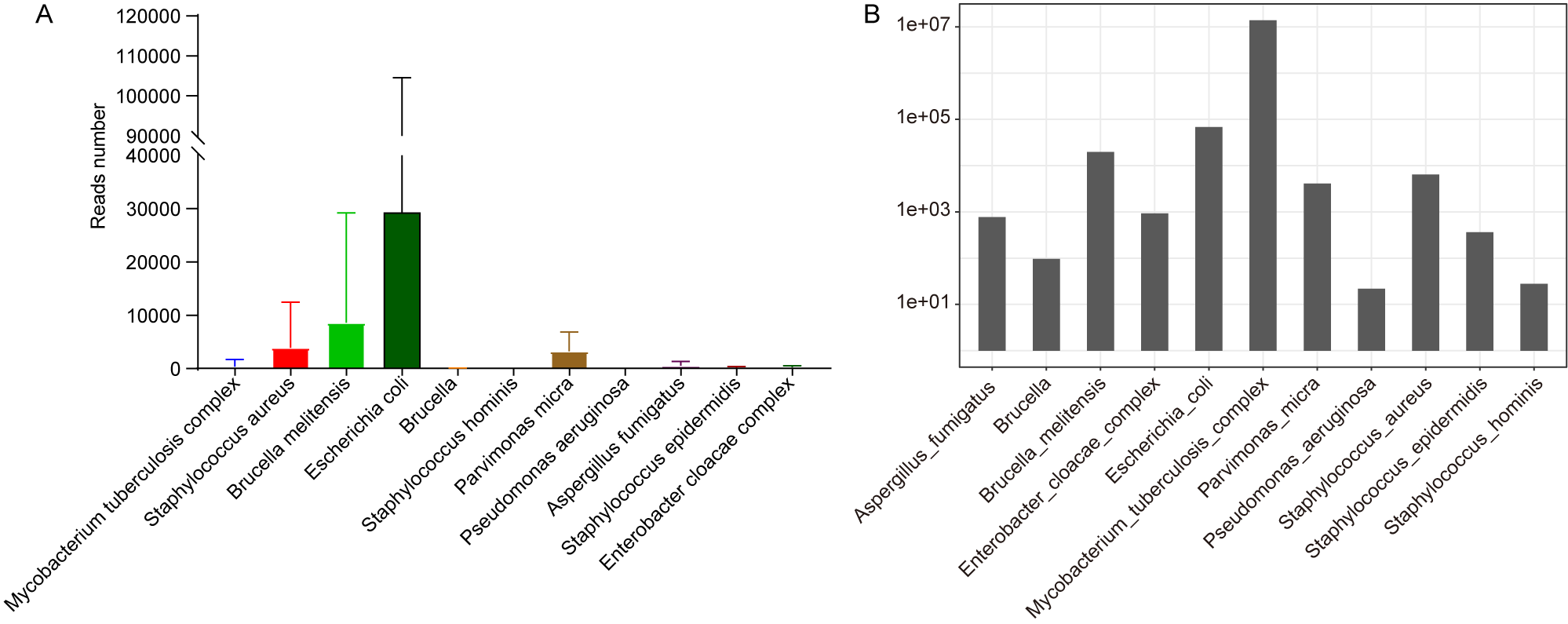

Supplement: Figure S4 — The read number and reads per million (RPM) of top 11 pathogens in 150 samples. [file spectrum.01064-24-s0004.tif]
