# Supplementary material for: Diagnostic value of metagenomic next-generation sequencing in patients with osteoarticular infections: a prospective study
Source: Microbiol Spectr. 2025 Apr 10;13(5):e01064-24. doi: 10.1128/spectrum.01064-24 (PMC12054075; doi:10.1128/spectrum.01064-24)
Supplement: Legends — for supplemental figures. [file spectrum.01064-24-s0005.docx]

**Figure S1 The pathogenic bacteria spectrum of primary (A) and invasive (B) osteoarticular infection.**

**Figure S2 Comparison of the type of organisms detected using the mNGS and CT methods in POI (A) and IOI (B) group.** POI: Primary osteoarthritis; IOI: Invasive osteoarthritis.

**Figure S3** Pathogens identified using both the mNGS and CT methods, mNGS only, and CT method only in POI (A) and IOI (B) groups. mNGS: metagenomic next-generation sequencing; CT: conventional tests. POI: Primary osteoarthritis; IOI: Invasive osteoarthritis.

**Figure S4 The read number and reads per million (RPM) of top 11 pathogens in 150 samples.**
